# Stepwise approach towards adoption of allergen immunotherapy for allergic rhinitis and asthma patients in daily practice in Belgium: a BelSACI-Abeforcal-EUFOREA statement

**DOI:** 10.1186/s13601-019-0243-1

**Published:** 2019-02-04

**Authors:** P. W. Hellings, B. Pugin, G. Mariën, C. Bachert, C. Breynaert, D. M. Bullens, J. L. Ceuppens, G. Clement, T. Cox, D. Ebo, P. Gevaert, S. Halewyck, V. Hox, K. Ladha, R. Jacobs, P. Rombaux, R. Schrijvers, K. Speleman, X. Van der Brempt, L. Van Gerven, O. Vanderveken, B. Verhaeghe, K. Vierstraete, S. Vlaminck, J. -B. Watelet, J. Bousquet, S. F. Seys

**Affiliations:** 1 ENT Clinical Department, University Hospital Leuven, Kapucijnenvoer 33, 3000 Louvain, Belgium; 2 Laboratory of Clinical Immunology, Department of Microbiology and Immunology, KU Leuven, Louvain, Belgium; 3 European Forum for Research and Education in Allergy and Airway Diseases, Brussels, Belgium; 4 ENT Clinical Department, University Hospital Ghent, Ghent, Belgium; 5 Upper Airways Research Laboratory, University of Ghent, Ghent, Belgium; 6 Internal Medicine Clinical Department, UZ Leuven, Louvain, Belgium; 7 Pediatrics Clinical Department, UZ Leuven, Louvain, Belgium; 8 ENT Clinical Department, AZ Damiaan, Ostend, Belgium; 9 ENT Clinical Department, Jessa Hospital, Hasselt, Belgium; 10 Immunology-Allergology-Rheumatology, University Hospital Antwerp, Antwerp, Belgium; 11 ENT Clinical Department, UZ Brussel, Brussels, Belgium; 12 ENT Clinical Department, ASZ Aalst, Aalst, Belgium; 13 ENT Clinical Department, Clinique Universitaires Saint-Luc, Brussels, Belgium; 14 Pediatrics Clinical Department, CHU Charleroi, Charleroi, Belgium; 15 ENT Clinical Department, AZ Sint-Blasius, Dendermonde, Belgium; 16 ENT Clinical Department, AZ Sint-Jan, Brugge, Belgium; 17 Allergopole, Clinique Saint-Luc Bouge, Namur, Belgium; 18 ENT Clinical Department, University Hospital Antwerp, Antwerp, Belgium; 19 ENT Clinical Department, St-Andries ziekenhuis, Tielt, Belgium; 20 ENT Clinical Department, AZ Groeninge, Kortrijk, Belgium; 21 ENT Clinical Department, AZ Delta Roeselare, Roeselare, Belgium; 22 Department of Respiratory Disease, University Hospital Arnaud de Villeneuve, Montpellier, France

**Keywords:** Allergic rhinitis, Asthma, Allergen-specific immunotherapy, Belgium

## Abstract

Allergic rhinitis (AR) affects 23–30% of the European population with equal prevalence reported in Belgium. Despite guidelines on the correct use of effective treatment, up to 40% of AR patients remain uncontrolled. Allergen immunotherapy (AIT) has been shown to improve the level of control up to 84% of patients being controlled by AIT. Recently, new guidelines for AIT have been published, supporting the clinical evidence for effectiveness of various subcutaneous and sublingual products for AIT in patients who are allergic to airborne allergens. AIT in AR patients not only reduces nasal and/or ocular symptoms but also induces tolerance and has preventive potential. Adoption of AIT into daily clinical practice in Belgium and other European countries is hampered primarily by reimbursement issues of each of the single products but also by several patient- and physician-related factors. Patients need to be better informed about the effectiveness of AIT and the different routes of administration of AIT. Physicians dealing with AR patients should inform patients on tolerance-inducing effects of AIT and are in the need of a harmonized and practical guide that supports them in selecting eligible patients for AIT, in choosing evidence-based AIT products and in following treatment protocols with proven efficacy. Therefore, a stepwise and holistic approach is needed for better adoption of AIT in the real-life setting in Belgium.

## Burden of allergic rhinitis

Allergic rhinitis (AR) represents one of the 3 phenotypic manifestations of rhinitis, besides infectious and non-allergic rhinitis [1, 2]. It is defined as the symptomatic inflammation of the nose induced by allergen inhalation in sensitized individuals [3]. Clinically, AR is characterized by symptoms of rhinorrhea, nasal obstruction, sneezing and itch. AR affects 23–30% of patients in Europe and thereby represents the most common non-communicable disease [4]. A recent Belgian study in over 2000 participants showed allergic sensitization in 40.3% of individuals with 30.9% also reported relevant symptoms of AR to the corresponding allergen [5]. Other studies in Belgium previously showed a prevalence of self-declared allergic rhinitis of 29% [6, 7] mirroring the European numbers. Symptomatic patients experience a significant impact of AR on their quality of life [8, 9]. Previous studies have shown that symptoms of AR significantly impact sleep [10], daily life [11] and in turn work productivity [12]. While missed work time (absenteeism), due to AR might be limited, over one-third of AR patients showed impaired work performance (presenteeism) according to a systematic analysis of 28 studies using the validated Work Productivity and Activity Impairment (WPAI) questionnaire [13]. Another study in over 1000 AR patients using the Allergy Diary mobile application confirmed work impairment in almost all patients with severe symptoms [14]. Finally, rhinitis has been identified as a prominent risk factor of new-onset asthma [15, 16]. AR is associated with an increased risk of uncontrolled asthma (adjusted odds ratio of 2.0), suggesting that proper control of the upper airways might reduce the burden of lower airway diseases [17].

## Socio-economic costs of AR

Because of its high prevalence in Belgium (30%) and Europe (23–30%), the socio-economic cost of AR is considerable. Total cost can be split in two categories, i.e. direct costs, which represent the expenses associated with medical resource utilization, and indirect cost, which are defined as the expenses from the work cessation or reduction of work productivity and missed opportunities in life. An American study revealed that AR might be the costliest disease of all from an employer perspective [18]. A recent EU-wide review showed that in Europe, avoidable indirect costs per patient insufficiently treated for allergy range between €55 and €151 billion annually due to absenteeism and presenteeism, corresponding to €2405 per untreated patient per year [19]. At the national level, a Swedish study estimated the total cost of AR at €1.3 billion annually [20], with 70% of costs due to presenteeism. No socio-economic studies have been conducted in Belgium. Nonetheless, one may extrapolate these findings to the Belgian patient population.

## Treatment algorithms for AR patients

Oral antihistamines alone or combined with intranasal corticosteroids (INS) are the cornerstone of AR treatment [3, 21]. Both treatment modalities reduce AR symptoms and burden of disease. Lately, the combination of azelastine and fluticasone propionate in a nasal spray has demonstrated clinical effectiveness along with a more rapid relief of symptoms when compared to INS alone in both adults and children with AR [22–24]. The positioning of these treatment modalities in relation to symptom severity has been published in ARIA guidelines [1, 25].

## Burden of uncontrolled disease in treated AR patients

Despite guideline-based treatment, uncontrolled disease is still observed in almost one-fifth of AR patients [26]. A more recent study in Belgium demonstrated that 37% of AR patients receiving currently available pharmacotherapy have uncontrolled disease [27]. Real-life data collected through mobile technology showed an evenly high percentage of patients with uncontrolled AR (unpublished data Allergy Diary). Different disease-, diagnosis-, patient- and treatment-related factors might be accountable for the high burden of uncontrolled disease [28, 29]. Remarkably, the prevalence of uncontrolled disease was only 16% in AR patients 3 years after initiation of subcutaneous allergen immunotherapy (AIT) [27]. This real-life study also showed that only 15% of AR patients attending a tertiary referral center for AR received AIT, illustrating the need for better adoption of AIT in daily practice.

**Fig. 1:**
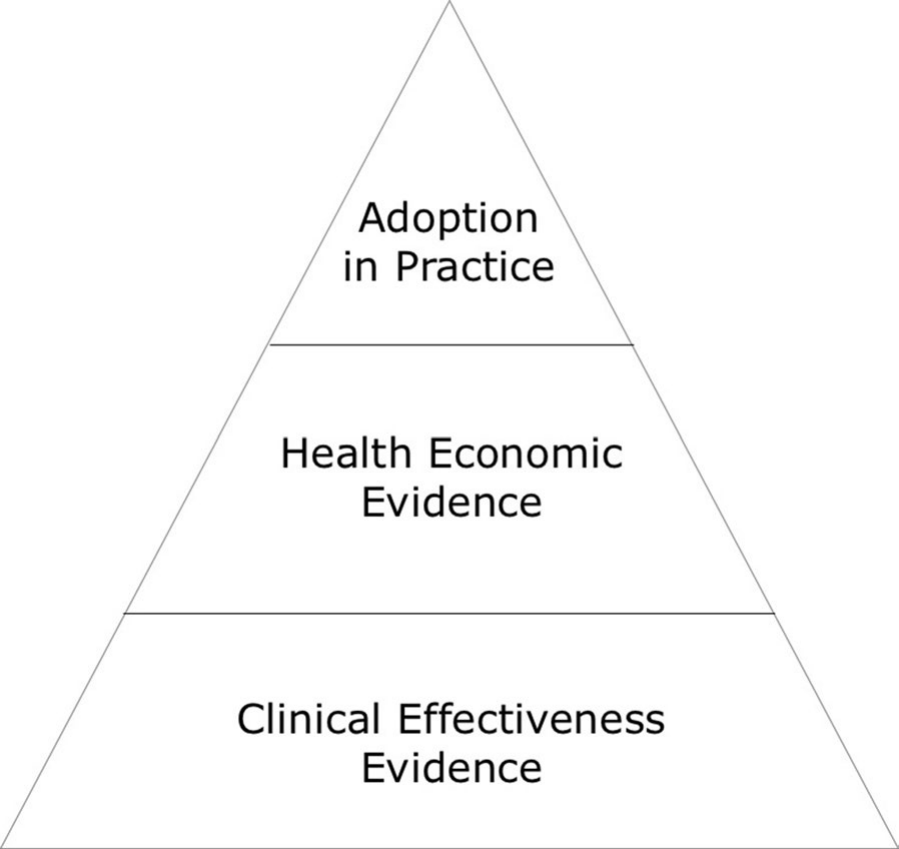
AIT adoption pyramid for real-life implementation

Three key milestones for AIT adoption have been identified and will be addressed here (Fig. 1). The first requirement relates to the clinical evidence on the treatment effectiveness and safety of AIT. The second requirement relates to the demonstration of cost-effectiveness—and thus health economic impact—of AIT, subsequently justifying reimbursement of the provided therapy. Finally, the third requirement relates to overcoming real-life barriers that impede AIT adoption in daily practice.

## Clinical evidence for AIT in the treatment of AR patients

According to an explorative patient survey conducted amongst newly diagnosed AR patients, 40% percent of Belgian AR patients expect a treatment that cures their disease [30]. AIT aims at inducing immunological tolerance and it is the only therapy for AR patients with disease modifying capacity [31]. In the ARIA guidelines, AIT is currently positioned as an additional treatment option for patients with uncontrolled disease despite adequate pharmacotherapy [25]. Both subcutaneous and sublingual immunotherapy (SCIT and SLIT) are available and the selection of the optimal therapeutic approach depends on the patients’ phenotype and endotype [32], the patients’ preference for SCIT or SLIT [30], the availability of specific AIT products with proven efficacy [33], and the (lack of) reimbursement of AIT [34].

Clinical evidence of AIT effectiveness and safety for allergic rhinoconjunctivitis was recently reviewed according to the Appraisal of Guidelines for Research and Evaluation (AGREE II) approach and resulted in a guideline document published by the European Academy for Allergy and Clinical Immunology (EAACI) [33, 35]. Meta-analysis of randomized controlled trials indicated that both SCIT and SLIT are effective for seasonal and perennial AR for its short-term benefit. Both are associated with reductions in symptoms and medication use. A treatment period of 3 years is recommended to achieve long-term efficacy persisting after treatment discontinuation. Recently, real-life evidence of birch-pollen AIT studies underlined the potential of AIT to induce long-term effects [31]. These studies showed reduced intake of AR and asthma medication and decreased risk of new-onset asthma medication use 6 years after cessation of AIT. Thus, broad evidence for the clinical efficacy of AIT has been demonstrated for AR but product-specific evaluation of evidence-based effectiveness is recommended [33].

In addition to the beneficial effect on nasal and ocular symptoms AIT has also been propagated for its effects on lower airways symptoms. A systematic review and meta-analysis led by an EAACI Task Force on AIT for allergic asthma concluded that substantial reductions in short-term symptom and medication scores were observed after AIT treatment [36]. More studies are needed to investigate the long-term effects on asthma. A recent study performed in children with grass pollen allergy demonstrated the potential of SLIT to prevent asthma symptoms and reducing asthma medication at 2 years of post-treatment follow-up [37]. More evidence is however needed to evaluate the preventive capacities of AIT in children, both with regard to development of new sensitizations as well as disease progression.

Finally, both SCIT and SLIT are safe and well-tolerated therapies when correctly applied [33]. SCIT injections should be given in a medical setting by experienced personnel trained in the early recognition and management of systemic reactions. A 4-year real-life US survey that included over 23.3 million injection visits reported systemic reactions in 0.1% of cases [38]. Systemic reactions with SLIT appear to be very unlikely although the overall rate of adverse reactions is similar between SCIT and SLIT. Other side effects include local reactions at the skin (redness, itching or swelling for SCIT), mouth (mucosal reactions for SLIT) or abdomen (abdominal pain for SLIT). Most of these reactions however occur during the initial phase of the treatment course and are considered to be of mild intensity and self-limiting [33].

## Evidence for cost-effectiveness of AIT in AR patients

Symptomatic treatment accounts for an important part of the direct and indirect costs of AR. Because of their sedating effects, first generation antihistamines impair mental performances of AR patients [39], thereby increasing indirect costs. Second generation antihistamines and intranasal antihistamines are effective and safe, without changing the course of the disease. INS are effective in reducing most of AR symptoms [40] but a significant number of patients fear adverse events [30].

The socio-economic evaluation of AIT needs to be seen in the context of cost savings caused by decreased consumption of symptomatic drugs, fewer visits to the GP and specialist, and increased work productivity. The 2010 revision of the ARIA guidelines called for further studies on the cost-effectiveness of AIT [21]. The quality-adjusted life-year (QALY) is a valuable health metric that encompasses the impact on both quantity and quality of life and is used to evaluate the cost-effectiveness of novel therapies. In a systematic review in 2017 funded by the EU, Asaria and coworkers have elegantly examined the cost-effectiveness studies of SLIT and SCIT versus standard care using QALY [41]. Nineteen studies from 14 European countries were analyzed (Germany, Denmark, Italy, UK, Austria, Finland, France, Sweden, the Netherlands, Canada, Czech Republic, Norway, Spain). Results from this systematic analysis revealed that both SLIT and SCIT can be considered as cost-effective in AR patients (with or without asthma) using the cost-effectiveness threshold of £20,000 (about €22 000) per QALY (as defined by the UK National Institute for Health and Care Excellence (NICE) [42], responsible for assessing health technology value for the National Health Service (NHS)). It is worth noting that discrepancies in terms of absolute values were observed between countries (due to the healthcare system in place), but all studies were below the threshold of €22 000 per QALY. Despite the economic evidence of cost-effectiveness, AIT is currently reimbursed in 56% of European countries (full reimbursement: 32% and partial reimbursement: 24%) [34]. In Belgium there is no reimbursement yet, which represents an obstacle towards immunotherapy adoption in clinical practice.

## Overcoming real-life barriers for AIT implementation in real-life

The first and second milestones for AIT adoption have been met, i.e. evidence for clinical efficacy and cost-effectiveness, through various European and Belgian studies. A consensus meeting organized by the National Institute for Health and Disability Insurance in Belgium (i.e. Rijksinstituut voor ziekte- en invaliditeitsverzekering, RIZIV) similarly concluded that sufficient clinical effectiveness is available and that cost-effectiveness is likely to be present after a minimal treatment with AIT of 3 years, especially given the reduction of other medical treatments and the long-term effects after cessation of the AIT [43].

**Fig. 2:**
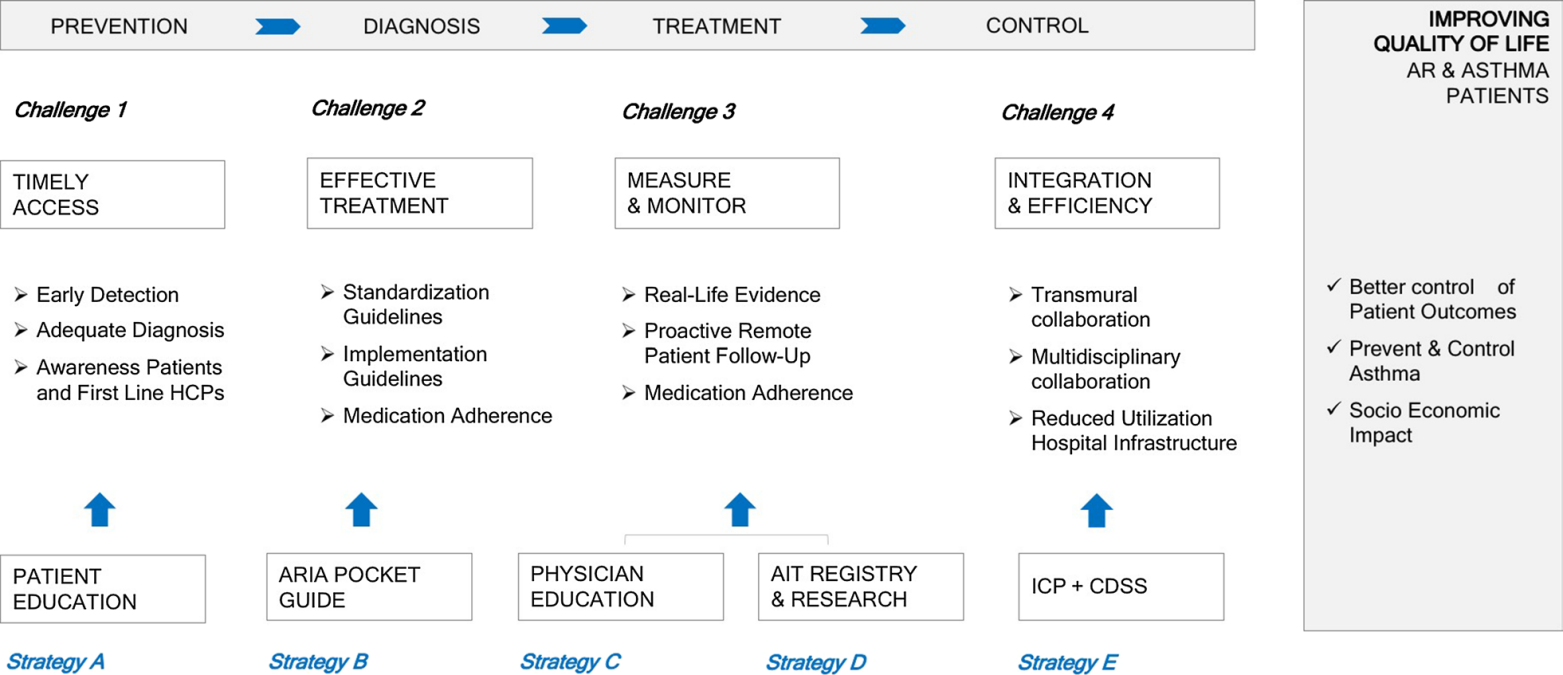
Overcoming real-life barriers for AIT adoption. AR: allergic rhinitis, CDSS: clinical decision support system, HCPs: health care professionals, ICP: integrated care pathway

Adoption of AIT in daily practice represents an additional and equally important milestone that requires relentless implementation focus, ultimately leading to better control of disease and prevention of asthma. The real-life barriers towards adoption of AIT in Belgium have been previously assessed in a nationwide survey amongst Ear-Nose-Throat (ENT) specialists and showed that the 2 main reasons for not prescribing AIT were lack of expertise by health care professionals (HCPs) and perception of high costs associated with AIT [44]. The latter barrier should be overcome by reimbursement of AIT in Belgium. Increasing the expertise and awareness on AIT requires education of physicians on how to select eligible patients for AIT, when to prescribe AIT, what product to prescribe and what protocol to follow, and how to follow up patients on AIT. Figure 2 summarizes 4 key challenges in care delivery for AR patients and the strategies to overcome these challenges in the context of improving the adoption of AIT in real-life.

The first challenge for adopting AIT in practice is to provide better, faster and more timely access of AR patients to the right treatment, including AIT. As such the burden of uncontrolled disease might be reduced and the preventive potential for asthma becomes reality. In that perspective, patients and HCPs need to be informed about the newest data on the efficacy and preventive potential of AIT, which will lead to increased awareness and hence will accelerate adequate diagnosis, early detection of good candidates for AIT, and timely referral to HCPs with specific interest in AIT. Patient Awareness Programs seem essential in order to improve patient and physician awareness. Interestingly, a nationwide multi-stakeholder public health campaign in Finland, so called ‘Finnish Allergy Program’ has been successfully implemented. Intermediate analysis after 5 years demonstrated clear improvements in clinical as well as health-economic outcomes of AR treatment [45]. On top of that, digital solutions, such as the Allergy Diary App and Patient E-Learning platforms can now be used in such campaigns as AR self-assessment tools to increase awareness but also to measure success of the campaign itself.

The second challenge for adopting AIT in practice can be overcome by following the AR guidelines and by increasing the adherence to AIT. In that perspective, global standardization, simplification and a more impactful implementation of AIT guidelines might be required, through practical education of physicians. Secondly, a drastic improvement of medication adherence is required and can be pursued through leverage of remote patient coaching and digital solutions [46]. Digital Tools, such as Clinical Decision Support Systems (CDSS) [47, 48] might be eligible to support HCPs in the adoption and application of AIT guidelines.

The third challenge relates to the need for real-life evidence on the impact of AIT on the quality of life and work productivity of AR patients. Continuous and remote monitoring of patients by digital technology will create big data on patient outcomes and can serve as a source for real-life evidence on AIT effects on AR (burden of AR and its comorbidities, impact of AIT, health-economic impact on AIT). Continuous monitoring furthermore enables a proactive and personalized approach of HCPs prior to and throughout the treatment and allows exchange of best practice protocols between HCPs. Additionally, it will also make sure we will better understand why patients respond to AIT and others don’t.

The fourth and last challenge relates to the day-to-day delivery of care. Optimization of the full care pathway of the patients can be achieved via better transmural (1st, 2nd and 3rd line of care) and multi-disciplinary (allergology, ENT, pulmonology, pediatrics) collaboration, which forms the basis of an integrated care pathway for patients with allergic rhinitis with or without asthma [49]. Consequently, this ensures that the right patient is exposed to the right therapy (in this case AIT) at the right moment. Moreover, it significantly improves efficiency, through a shorter length of the entire pathway, alignment on necessary and priority patient visits, and improved utilization of infrastructure.

## Conclusion and recommended next steps

**Fig. 3:**
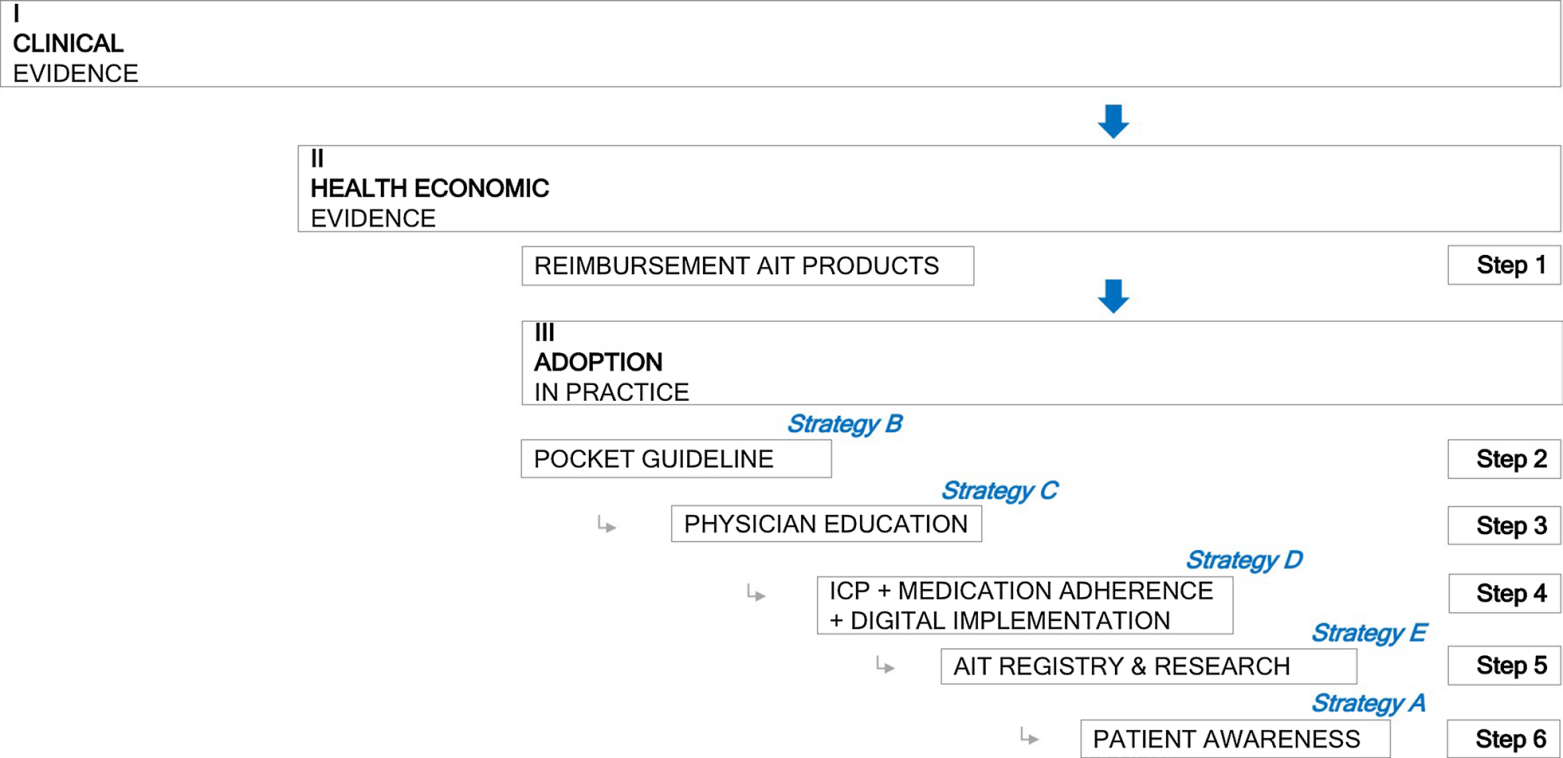
ARIA-EUFOREA implementation strategy for AIT adoption. ICP: integrated care pathway

Clinical efficacy and safety data support the recommendation of SCIT and SLIT for use in patients with AR. However, physicians should look into product-specific evidence before prescribing AIT to patients and they should follow treatment protocols for AIT products with proven efficacy. Health-economic evaluation in Europe has provided evidence for cost-effectiveness of AIT. However, cost-effectiveness evaluation studies in Belgium are lacking and are needed to move forward towards better implementation and reimbursement of AIT in Belgium. Further adoption of AIT into daily practice will require a stepwise approach with active engagement of all stakeholders involved across the full care pathway of the AR patient (Fig. 3). The subsequent steps which are required are listed here.

### Step 1

Reimbursement of specific AIT products in Belgium. Reimbursement procedures however might take time. The effects of adoption of in practice (steps 2–6) will grow once step 1 is met but should be initiated as soon as possible.

### Step 2

Develop and disseminate a simple AIT Pocket Guide for physicians to better understand and prescribe AIT.

### Step 3

Provide practical and evidence-based education for physicians through national or regional Workshops or Masterclasses for change management in AIT.

### Step 4

Implement Integrated Care Pathways supported by the development and deployment of a Clinical Decision Support System (CDSS).

### Step 5

Implement a national registry for AIT in hospitals and private allergy clinics.

### Step 6

Deploy Patient Awareness Programs based on self-management proposals of next-generation care pathways including ARIA-EUFOREA educational materials.
